# Timosaponin AIII Inhibits Migration and Invasion Abilities in Human Cervical Cancer Cells through Inactivation of p38 MAPK-Mediated uPA Expression In Vitro and In Vivo

**DOI:** 10.3390/cancers15010037

**Published:** 2022-12-21

**Authors:** Hung-Ju Chien, Chung-Jung Liu, Tsung-Ho Ying, Pei-Ju Wu, Jiunn-Wei Wang, Yi-Hsuan Ting, Yi-Hsien Hsieh, Shih-Chiang Wang

**Affiliations:** 1 Institute of Medicine, Chung Shan Medical University, Taichung 40201, Taiwan; 2 Department of Obstetrics and Gynecology, Changhua Christian Hospital, Changhua 50006, Taiwan; 3 Division of Gastroenterology, Department of Internal Medicine, Kaohsiung Medical University Hospital, Kaohsiung 807378, Taiwan; 4 Regenetative Medicine and Cell Therapy Research Center, Kaohsiung Medical University, Kaohsiung 807378, Taiwan; 5 Department of Obstetrics and Gynecology, Chung Shan Medical University Hospital, Taichung 40201, Taiwan; 6 Department of Obstetrics and Gynecology, School of Medicine, Chung Shan Medical University, Taichung 40201, Taiwan; 7 Department of Medicine, Faculty of Medicine, College of Medicine, Kaohsiung Medical University, Kaohsiung 807378, Taiwan; 8 Department of Medical Research, Chung Shan Medical University Hospital, Taichung 40201, Taiwan; 9 Department of Obstetrics and Gynecology, Chung-Kang Branch, Cheng Ching Hospital, Taichung 40764, Taiwan

**Keywords:** Timosaponin AIII, human cervical cancer, metastasis, p38 MAPK, uPA

## Abstract

**Simple Summary:**

Cervical cancer is one of the most common cancers in women. High resistance to chemotherapeutic agents and adaptation to radiotherapy in cervical cancer patients can lead to severe mortality. The aim of our study was to assess Timosaponin AIII (TSAIII) as a novel therapeutic approach for cervical cancer. Our findings first revealed that TSAIII suppresses cell migration, cell invasion, p38 activation and uPA expression. The fact that p38 knockdown synergistically contributes to TSAIII-inhibited uPA expression and invasion activity reveals that the p38–uPA axis mediates the anticancer properties of TSAIII. The antimetastatic properties of TSAIII were further confirmed through findings of pulmonary metastasis in immunodeficient mice in vivo and the anticancer stemness property of TSAIII in cervical cancer stem cells (CCSCs) in vitro.

**Abstract:**

Cervical cancer is one of the most common gynecologic cancers globally that require novel approaches. Timosaponin AIII (TSAIII) is a steroidal saponin that displays beneficial effects in antitumor activities. However, the effect of TSAIII on human cervical cancer remains unknown. In this study, we found that TSAIII showed no influence on cell viability, cytotoxicity, cell cycle distribution and apoptosis induction in human cervical cancer cells. TSAIII was revealed to have a significant inhibitory effect on cell migration and invasion through the downregulation of invasion-related uPA expression and p38 MAPK activation in both human cervical cancer cells and cervical cancer stem cells (CCSCs), indicating that the p38 MAPK–uPA axis mediated the TSAIII-inhibited capacity of cellular migration and invasion. In a synergistic inhibition assay, a TSAIII plus p38 siRNA cotreatment revealed a greater inhibition of uPA expression, migration and invasion in human cervical cancer cells. In an immunodeficient mouse model, TSAIII significantly inhibited lung metastases from human cervical cancer SiHa cells without TSAIII-induced toxicity. These findings first revealed the inhibitory effects of TSAIII on the progression of human cervical cancer through its downregulation of p38 MAPK–uPA axis activation. Therefore, TSAIII might provide a potential strategy for auxiliary therapy in human cervical cancer.

## 1. Introduction

Cervical cancer is one of the most common cancers in women. According to updated databases from the World Health Organization (WHO), an estimated 640,000 women were diagnosed with cervical cancer globally, and 324,000 women died from this disease in 2020 [1]. The recurrence and poor prognosis of cervical cancer primarily result in death, although treatments such as surgery, chemotherapy, radiation therapy and even a combination of these strategies are used for cervical cancer [2]. High resistance to chemotherapeutic agents and adaptation to radiotherapy in cervical cancer patients can lead to severe mortality [3]. Therefore, there is an urgent need to identify novel therapeutic approaches for cervical cancer.

Metastasis is an important aspect of cervical cancer prognosis and target therapy. Metastasis deteriorates tumor progression through the remodeling of the extracellular matrix (ECM), which promotes tumor cell migration and invasion from the primary tumor site to the target organ [4]. The metastasis of cervical cancer cells to other parts of the body, such as lymph nodes [5], lung [6], bone [7], liver [8] and bowel [9], acts as the critical factor that results in the high mortality rate in cervical cancer patients. In the proteolytic proteinase systems, the urokinase-type plasminogen activator (uPA) is a key factor responsible for ECM degradation [4,10]. Dihydromyricetin-inhibited uPA contributes to the downregulation of osteosarcoma metastasis through SP-1 and NF-κB modifications [11]. uPA downregulation due to synergistic effects of licochalcone plus sorafenib significantly suppresses human SK-Hep-1 hepatocellular carcinoma (HCC) cell-mediated lung metastasis [12]. uPA is shown to contribute to pentraxin-3-induced tumorigenesis and metastasis in human cervical cancer cells [10].

Natural phytochemicals are still widely investigated for their efficacy against cancers. Steroidal saponins are the major compounds of *Anemarrhena asphodeloides*, which are shown to display biological activities such as anti-inflammatory, antiproliferative, antimetastatic, antiangiogenic and anti-multidrug-resistance effects in in vitro and in vivo studies [13,14]. Timosaponin AIII (TSAIII) is one type of steroidal saponin and is demonstrated to possess proapoptotic and antimetastatic effects in melanoma, colorectal carcinoma, breast cancer, non-small-cell lung cancer and osteosarcoma [15,16,17,18,19]. However, it is still unknown if TSAIII can suppress cervical cancer progression. In the present study, TSAIII was demonstrated to inhibit cell migration and invasion through the downregulation of the p38 MAPK–uPA axis in both human cervical cancer cells and cervical cancer stem cells (CCSCs) in vitro and in vivo.

## 2. Materials and Methods

### 2.1. Chemical Reagents and Antibodies

Timosaponin AIII (TSAIII; CFN98151) was obtained from ChemFaces (Wuhan, Hubei, PRC). The DMSO, MTT and isopropanol were obtained from Merck KGaA (Darmstadt, Germany). Primary antibodies against uPA, total-ERK, phospho-JNK, total-JNK, phospho-p38MAPK, total-p38MAPK, uPA, GAPDH and siRNA-p38 were purchased from Santa Cruz Biotechnology (Dallas, TX, USA). The antibody of phospho-ERK, SOX2, Nanog, OCT4 and CD49f were purchased from Cell Signaling Technology (Danvers, MA, USA).

### 2.2. Cell Culture

The human cervical cancer cell lines HeLa and C33A were purchased from the Bioresources Collection and Research Center (Hsinchu, Taiwan). Human normal cervical epithelial (CerEpi) cells and human cervical cancer cell lines SiHa and CaSki were obtained from the American Type Culture Collection (Rockville, MD, USA). HeLa cells were cultured in Dulbecco′s Modified Eagle′s Medium/Nutrient Mixture F-12 Ham (DMEM/F-12) medium (Invitrogen, Carlsbad, CA, USA), C33A and SiHa were cultured in Dulbecco’s modified Eagle’s Medium (DMEM) (Invitrogen), and CaSki cells were maintained in RPMI-1640 medium. These cells were supplemented with 2 mM of glutamine, 100 U/mL of penicillin and 100 µg/mL of streptomycin (Sigma), in addition to 10% heat-inactivated fetal bovine serum (FBS; HyClone, Logan, UT, USA). CerEpi cells were cultured with Cervical Epithelial Cell Basal Medium (ATCC PCS-480-032). To make the complete medium, the contents of the Cervical Epithelial Cell Growth Kit (ATCC PCS-480-042) were added to the basal medium. Cultures were maintained at 37 °C in a humidified atmosphere with 5% CO_2_. Cells were passaged every 2 days to obtain exponential growth. For the CCSC culture, as previously described [20], SiHa CCSCs were cultured in DMEM/F12 medium (0.4% bovine serum albumin (BSA), 10 ng/mL of epidermal growth factor, 10 ng/mL of basic fibroblast growth factor and 5 μg/mL of insulin) in ultra-low-adherence dishes and six-well plates (Merck KGaA, Darmstadt, Germany) for 7 days. Sphere formation was monitored daily.

### 2.3. Western Blotting Analysis

Human cervical cancer cells were treated with different concentrations of TSAIII for 24 h. Cells were lysed in NETN buffer with protease inhibitors in ice for 15 min and then were collected, and the total proteins were quantified. Then, 20 μg of total protein from each group was separated using 10% SDS–PAGE and transferred onto a PVDF membrane for 70 min. The PVDF membrane was incubated with 5% nonfat dry milk in a TBST buffer for 1 h, including a 0.1% Tween-20 solution. Next, the primary antibodies against uPA (1:1000), phospho-ERK (1:1000), total-ERK (1:1000), phospho-JNK (1:1000), total-JNK (1:1000), phospho-p38 (1:1000), total-p38 (1:1000), SOX2 (1:1000), Nanog (1:1000), CD49f (1:1000), OCT4 (1:1000) and GAPDH (1:5000) were incubated with the membrane for 1 h, after washing with the TBST buffer three times. Subsequently, the membrane incubated with secondary anti-rabbit (1:10,000) or anti-mouse (1:10,000) antibodies for measuring antibody-bound protein bands was visualized with an enhanced chemiluminescence buffer and analyzed with a LAS 4000 mini analyzer (GE Healthcare Uppsala, Sweden). The relative protein expression was calculated as a % of the control (untreated cells).

### 2.4. Cell Growth Assay

The cell growth ability of human cervical cancer and normal cervical epithelial (CerEpi) cells treated with TSAIII was detected by using an MTT assay. Cells (8 × 10^3^/well) were seeded in 24-well culture plates, and different concentrations of TSAIII were added for 24 h. The MTT (0.5 mg/mL) reagent was added to cells treated with or without TSAIII for 4 h. Then, the MTT reagent was removed, and 0.5 mL of isopropyl alcohol was added, which was measured at 470 nm by using a Multiskan MS ELISA reader (Labsystems, Helsinki, Finland).

### 2.5. Three-Dimensional Sphere Formation

Cells were suspended at a density of 5000 cells/mL in serum-free DMEM/F12 containing 100 IU/mL of penicillin, 100 µg/mL of streptomycin, 20 ng/mL of human recombinant epidermal growth factor, 10 ng/mL of human recombinant basic fibroblast growth factor, 2% B27 and 1% N2 supplement. Then, the cells were seeded into ultra-low-attachment 6-well plates. Suspension cultures were carried out for 7 days until 3D spheres were formed.

### 2.6. Analysis of Cell Cycle and Cell Apoptisis 

For the PI staining assay, human cervical cancer cells were treated with TSAIII (0, 2, 4 and 6 μM) for 24 h, and cells were digested with 0.25% trypsin (EDTA-free) and fixed with 70% chilled ethanol for 2 days, then stained with the propidium iodide (PI) reagent for 15 min. The DNA content was detected by using the Muse Cell Analyzer to conduct flow cytometry (Merck Millipore, Burlington, MA, USA), whereas the percentage of cell cycle distribution was calculated with the Muse^®^ Cell Analyzer (Merck Millipore, Burlington, MA, USA). Cell apoptosis was evaluated after the treatment of TSAIII with human cervical cancer cells using the Muse Annexin V and Dead Cell kit (Merck Millipore, Burlington, MA, USA) for 15 min at room temperature, whereas the percentage of nonapoptotic or apoptotic cells was calculated by using the Muse^®^ Cell Analyzer to conduct flow cytometry (Merck Millipore, Burlington, MA, USA).

### 2.7. In Vitro Migration and Invasion Assay 

In vitro migration and invasion were analyzed according to the procedure reported previously [21]. The treatment of cervical cancer cells with TSAIII was carried out for 24 h, and then, cells (1 × 10^5^/well) were added onto the polycarbonate membranes (8 μm pores) that were precoated with or without a Matrigel matrix (1:10) for cell invasion assay and migration assay, respectively. After 16 h (migration assay) and 24 h (invasion assay), cells migrating or invading the lower side of the membrane were fixed with methanol for 10 min and stained with the Giemsa reagent (1:20) for 30 min. Migrative cell numbers were counted for three random positions via microscope (200× magnification). Each experiment was repeated three times.

### 2.8. Total RNA Extraction and Real-Time PCR Assay

Total RNA was isolated from cultured cells as previously described [22]. The cells were homogenized in an RNA lysis/binding buffer. The High Pure RNA Tissue Kit (Roche Applied Science, Mannheim, Germany) was used for RNA extraction. The standard reverse transcription and real-time PCR protocol, as previously described, was used in this study [22]. For reverse transcription, the samples were incubated at 25 °C for 10 min; real-time PCR was initiated with a hot start (10 min at 95 °C, 1 cycle); the samples were then subjected to 40 cycles at 95 °C for 15 sec and 60 °C for 1 min. Data were analyzed by StepOne real-time PCR system (Applied Biosystems, Foster City, California, USA). Primers were as follows: human uPA forward primer 5′-TTGCGGCCATCTACAGGAG-3′, reverse primer 5′- ACTGGGGATCGTTATACATC-3′; human SOX2 forward primer 5′- CGAGTGGAAACTTTTGTCGGA-3′; reverse primer 5′- TGTGCAGCGCTCGCAG-3′; human OCT4 forward primer 5′- GTGGAGAGCAACTCCGATG-3′, reverse primer 5′-TGCTCCAGCTTCTCCTTCTC-3′; human Nanog forward primer 5′-ATTCAGGACAGCCCTGATTCTTC-3′; 5′-TTTTTGCGACACTCTTCTCTGC-3′; human CD49f forward primer 5′-CGAAACCAAGGTTCTGAGCCCA-3′; 5′-CTTGGATCTCCACTGAGGCAGT-3′; human GAPDH forward primer 5′- CGGAGTCAACGGATTTGGTCGTAT-3′ reverse primer 5′-AGCCTTCTCCATGGTGGTGAAGAC-3′ (MISSION BIOTECH, Taipei, Taiwan). Relative gene expression was obtained after normalization with endogenous GAPDH and determination of the difference in threshold cycle (Ct) between treated and untreated cells using 2^−ΔΔCt^ method.

### 2.9. Lung Metastasis Animal Experiment

SiHa cells (1.5 × 105 cells/0.1 mL of PBS) were intravenously injected into the tail vein of immunodeficient female BALB/c nude mice. After seven days, the mice were randomly divided into three groups, one which was untreated (control group, n = 5), and the other two which were orally gavaged with 5 mg/kg of TSAIII (n = 5) and 10 mg/kg of TSAIII (n = 5) twice per week for 1 month. After finishing the study, the mice were euthanized with CO_2_, and the tissues were collected and fixed in 10% formalin. All tissue sections (lung, kidney, heart, spleen and liver) were stained with hematoxylin and eosin (H&E) for morphological analysis and safety evaluation. The immunohistochemistry assay was used to observe Ki-67 expression (cell proliferative marker) in the TSAIII-treated lung tissue group under an optical microscope. All the animal experiments were performed according to the Guide for the Care and Use of Laboratory Animals of the Council of Agriculture, Executive Yuan, Taiwan and accredited by the Institutional Animal Care and Use Committee (IACUC) of Chung Shan Medical University, Taiwan (IACUC number-2523).

### 2.10. Statistical Analysis

Each experiment was repeated at least three times. Results were presented as the mean ± SE, and statistical comparisons were made using Student’s *t*-test. Significance was defined at the level of *p* < 0.05 or 0.01.

## 3. Results

### 3.1. Effect of TSAIII on Cell Viability and Cytotoxicity in Human Cervical Cancer Cells

**Figure 1:**
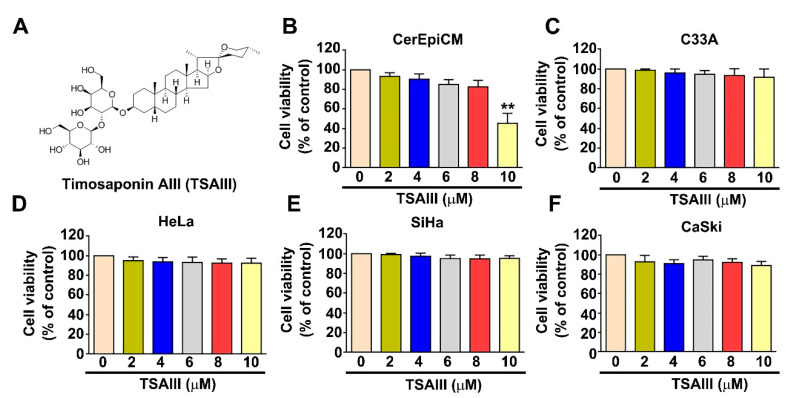
Effect of TSAIII on viability and cytotoxicity of human cervical cancer cells. (**A**) Structure of Timosaponin AIII (TSAIII). (**B**) CerEpi cells (human normal cervical epithelial cells). (**C**–**F**) C33A, HeLa, SiHa and CaSki cells (human cervical cancer cells) were exposed to various concentrations (0, 2, 4, 6, 8 and 10 μM) of TSAIII for 24 h and then measured via MTT assay to observe cell viability (mean ± SE, n = 3). ** *p* < 0.01.

The structure of Timosaponin AIII (TSAIIIA) is presented in Figure 1A. The effect of TSAIII on cell viability and cytotoxicity was analyzed in normal human CerEpi cells and human cervical cancer cells, including C33A, HeLa, SiHa and CaSki cells. These cell lines were exposed to various concentrations (0, 2, 4, 6, 8 and 10 μM) of TSAIII for 24 h and then were measured by using an MTT assay. The influence of TSAIII (≤8 μM) on cell viability or cytotoxicity induction was not observed in human normal CerEpi cells (Figure 1B) and four human cervical cancer cells (Figure 1C–F); only the highest amount of TSAIII, 10 μM, could decrease the cell viability of human CerEpi cells to approximately 50% of the control-treated cells (Figure 1B). Therefore, we chose TSAIII concentrations of 2, 4 and 6 μM to be used for further cell experiments.

### 3.2. Effect of TSAIII on Cell Cycle Distribution and Apoptosis Induction in Human Cervical Cancer Cells

**Figure 2:**
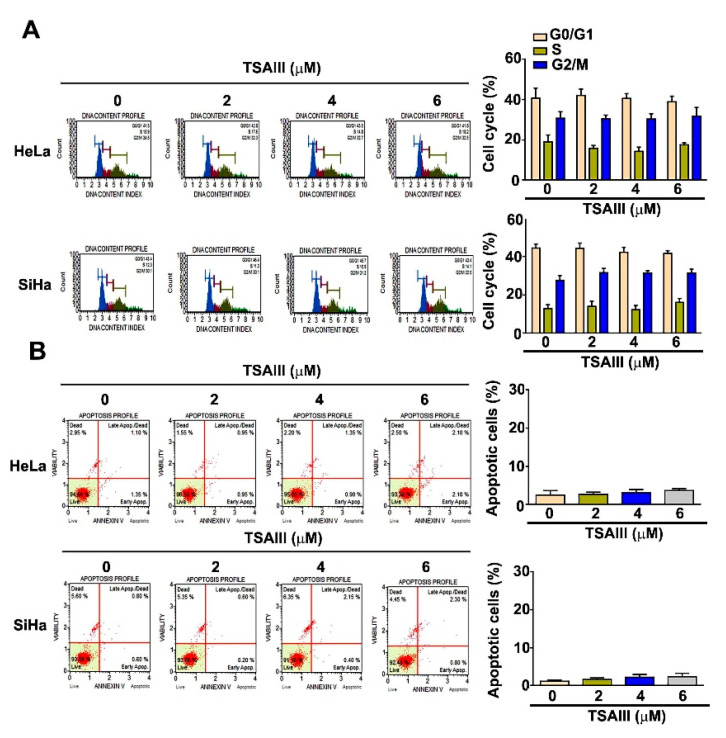
Effect of TSAIII on cell cycle arrest and apoptosis induction in human cervical cancer cells. (**A**,**B**) Regulation of cell cycle distribution and cell apoptosis in human HeLa and SiHa cervical cancer cells exposed to various concentrations (0, 2, 4 and 6 μM) of TSAIII was analyzed via flow cytometry (mean ± SE, n = 3).

Human HeLa and SiHa cervical cancer cells were exposed to various concentrations (0, 2, 4 and 6 μM) of TSAIII for 24 h, which was then followed by a flow cytometry analysis. The TSAIII treatment showed no influence on cell arrest at any phase (Figure 2A). Induction of cell apoptosis in HeLa and SiHa cells was also not observed through the flow cytometry analysis (Figure 2B). The results showed that TSAIII has no effect on the induction of cell cycle arrest and apoptosis in these human cervical cancer cells. 

### 3.3. TSAIII Inhibits Cell Migration and Invasion in Human Cervical Cancer Cells

**Figure 3:**
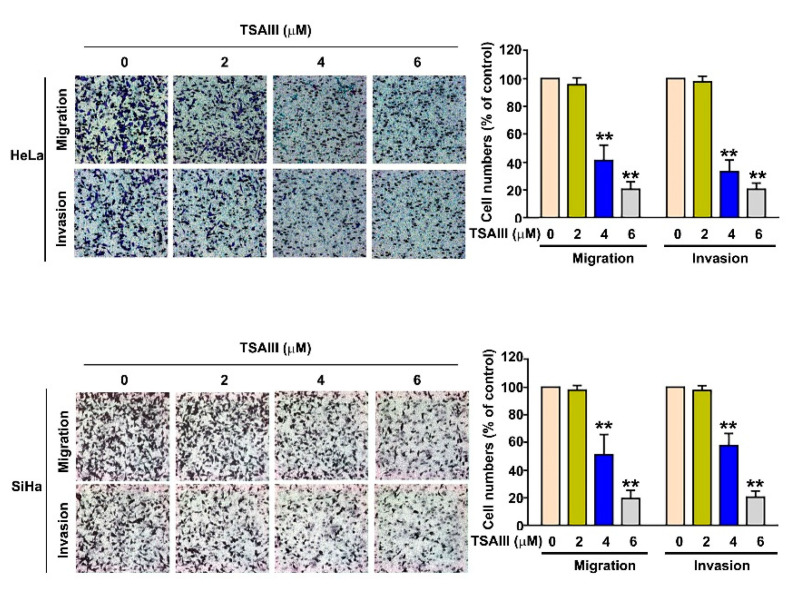
Effect of TSAIII on cell migration and invasion in human cervical cancer cells. Human HeLa and SiHa cervical cancer cells were exposed to various concentrations of TSAIII (0, 2, 4 and 6 μM) for 24 h, which was then followed by measuring the capacity of cellular migration for 16 h and invasion for 24 h. ** *p* < 0.01 versus control (line 1) (mean ± SE, n = 3).

To investigate the effect of TSAIII on the capacity of cellular migration and invasion in human cervical cancer cells, we treated HeLa and SiHa cells with various concentrations of TSAIII (0, 2, 4 and 6 μM) for 24 h and carried out the assays to observe cellular migration and invasion. TSAIII was shown to significantly inhibit cellular migration and invasion activity in both human HeLa and SiHa cells in a dose-dependent manner (Figure 3).

### 3.4. TSAIII Inhibits the uPA Expression in Human Cervical Cancer Cells

**Figure 4:**
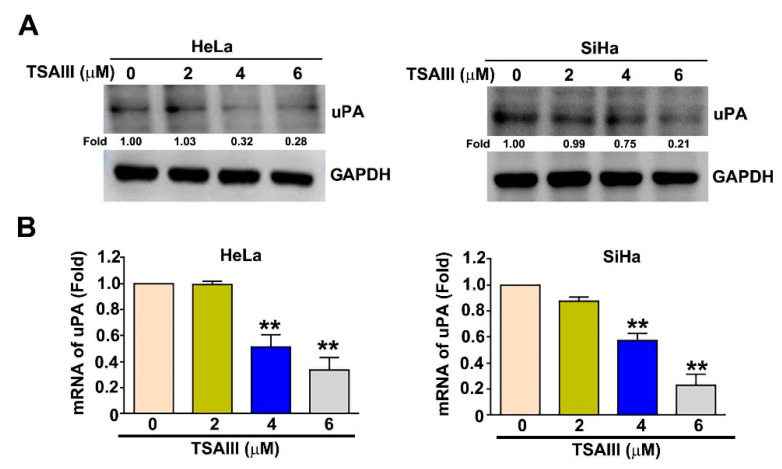
TSAIII regulation of uPA expression of human cervical cancer cells. Human HeLa and SiHa cervical cancer cells were treated with various concentrations of TSAIII (0, 2, 4 and 6 μM) for 24 h and then harvested to detect protein and mRNA levels of uPA through immunoblotting (**A**) and RT-qPCR (**B**) assays. GAPDH as internal control. ** *p* < 0.01 versus control (line 1) (mean ± SE, n = 3). Uncropped WB images were shown in Figure S1.

The urokinase-type plasminogen activator (uPA) contributes to cytoskeletal remodeling, migration and invasion in tumor progression [23]. Therefore, human HeLa and SiHa cells exposed to different concentrations of TSAIII (0, 2, 4 and 6 μM) were analyzed via immunoblotting and RT-qPCR assays to investigate the effect of TSAIII on uPA expression. We observed that TSAIII decreased the protein and mRNA expression of uPA in HeLa and SiHa cells (Figure 4A,B).

### 3.5. TSAIII Inhibits the Activation of p38 MAPK Pathway in Human Cervical Cancer Cells 

**Figure 5:**
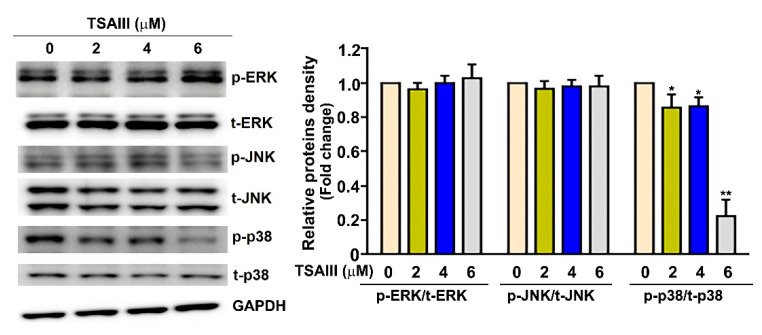
TSAIII downregulates the p38 MAPK signaling pathway in human SiHa cervical cancer cells. Human SiHa cervical cancer cells were treated with TSAIII (0, 2, 4 and 6 μM) for 24 h. SiHa cells were then harvested for use in immunoblotting assay to observe the activation of signaling pathway. GAPDH as internal control. * *p* < 0.05 ** *p* < 0.01 versus control (line 1) (mean ± SE, n = 3). t: total; p: phosphoryled. Uncropped WB images were shown in Figure S1.

To investigate which signal transduction pathway(s) was involved in the mechanism behind the downregulated capacity of TSAIII in the migration and invasion in human cervical cancer cells, we treated human SiHa cervical cancer cells with TSAIII (0, 2, 4 and 6 μM). Then, SiHa cells were harvested for use in the immunoblotting assay to measure the activation of the signaling pathway. TSAIII was shown to reduce the phosphorylation of the p38 MAPK (p-p38) signaling pathway (Figure 5). The results indicate that the p38 MAPK signaling pathway is involved in the TSAIII-inhibited invasion activity in human cervical cancer cells.

### 3.6. p38 MAPK Pathway Involved in TSAIII Inhibits uPA Expression, Cell Migration and Invasion in Human Cervical Cancer Cells

**Figure 6:**
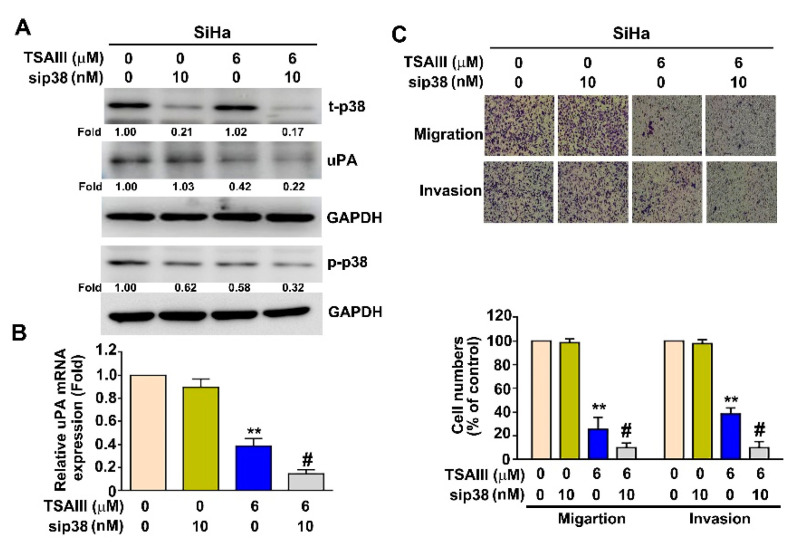
Cotreatment inhibitory effect of TSAIII and p38 siRNA on uPA expression, migration and invasion in human cervical cancer cells. (**A**) Human SiHa cervical cancer cells were treated with various concentrations of TSAIII (0 and 6 μM) and/or p38 siRNA (0 and 10 nM), and then were harvested for the detection of the expression of t-p38 (total-p38), p-p38 (phosphoryled-p38) and uPA via immunoblotting assay. (**B**) The mRNA level of uPA was determined with RT-qPCR assay. (**C**) The capacity of migration and invasion of human SiHa cervical cancer cells was analyzed after TSAIII treatment in the presence or absence of p38 siRNA (10 nM) for 24 h. ** *p* < 0.01 versus control (line 1); # *p* < 0.05 versus TSAIII alone treatment (line 3) (mean ± SE, n = 3). t: total; p: phosphoryled. Uncropped WB images were shown in Figure S1.

To identify the p38 MAPK pathway’s involvement in the TSAIII inhibition of uPA expression and cell metastasis in human SiHa cells, SiHa cells were exposed to TSAIII (0 and 6 μM) and/or p38 siRNA (0 and 10 nM). It was found that the TSAIII (6 μM) plus p38 siRNA (10 nM) cotreatment significantly showed a greater inhibitory effect on p-p38 p-p38 and uPA protein (Figure 6A) and uPA mRNA expression (Figure 6B). Interestingly, this cotreatment of TSAIII (6 μM) and p38 siRNA (10 nM) revealed the inhibition of cellular migration and invasion in human SiHa cells (Figure 6C).

### 3.7. TSAIII Inhibits Cancer Stemness, p38 Activation, uPA Expression and Invasion in Human Cervical Cancer Stem Cells 

**Figure 7:**
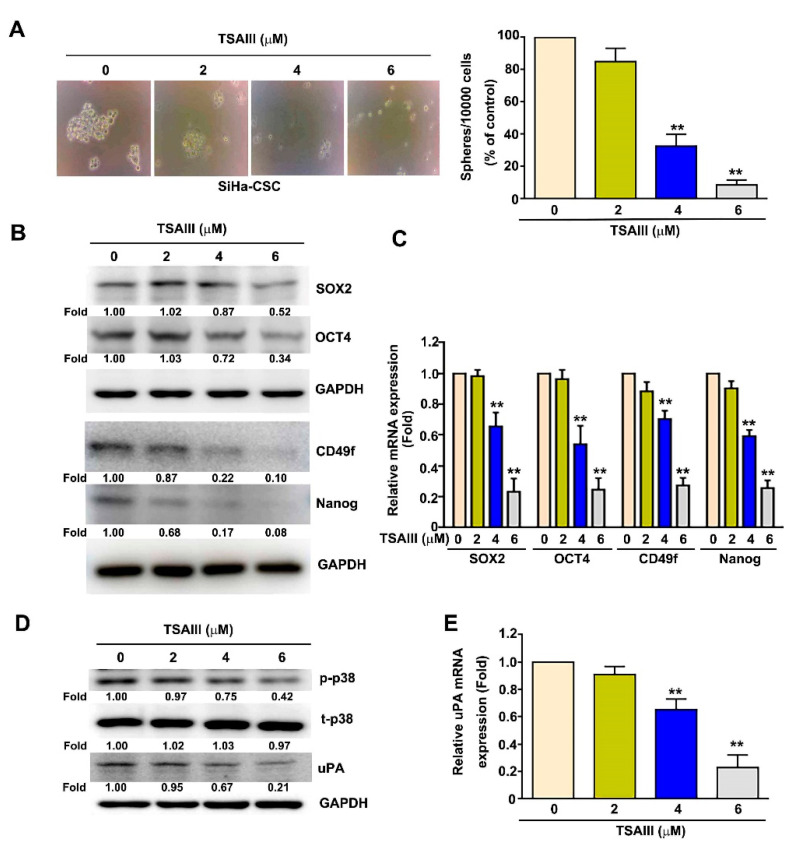
TSAIII regulation of cancer stemness, p38 MAPK activation and uPA expression in human cervical cancer stem cells. Human SiHa cervical cancer stem cells were treated with various concentrations of TSAIII (0, 2, 4 and 6 μM) for 24 h and then harvested to observe the 3D sphere formation (**A**) and detect the expression of stemness factors SOX2, OCT4, Nanog and CD49f via immunoblotting (**B**) and RT-qPCR (**C**) assays. (**D**,**E**) After TSAIII (0, 2, 4 and 6 μM) treatment, t-p38 (total-p38), p-p38 (phosphoryled-p38) and uPA expression were then measured via immunoblotting and RT-qPCR assays. GAPDH as internal control. ** *p* < 0.01 versus control (line 1) (mean ± SE, n = 3). Uncropped WB images were shown in Figure S1.

To investigate the effect of TSAIII on cancer stemness activation, the ability to form 3D spheres was analyzed in human SiHa cervical cancer stem cells (CCSCs). After treatment with various concentrations of TSAIII (0, 2, 4 and 6 μM) for seven days, TSAIII was shown to significantly inhibit 3D sphere formation in human SiHa CCSCs in a dose-dependent manner (Figure 7A). The regulating factors of cancer stemness were further measured, which indicated that TSAIII significantly downregulated the protein and mRNA expressions of SOX2, OCT4, Nanog and CD49f in a dose-dependent manner (Figure 7B,C). Activation of p38 MAPK and the expression of uPA were further confirmed in TSAIII-treated SiHa CCSCs. We observed that TSAIII significantly inhibited p38 MAPK activity (Figure 7D) and decreased the protein and mRNA levels of uPA in SiHa CCSCs (Figure 7D,E).

### 3.8. TSAIII Inhibits Lung Metastasis of Human Cervical Cancer Cells

**Figure 8:**
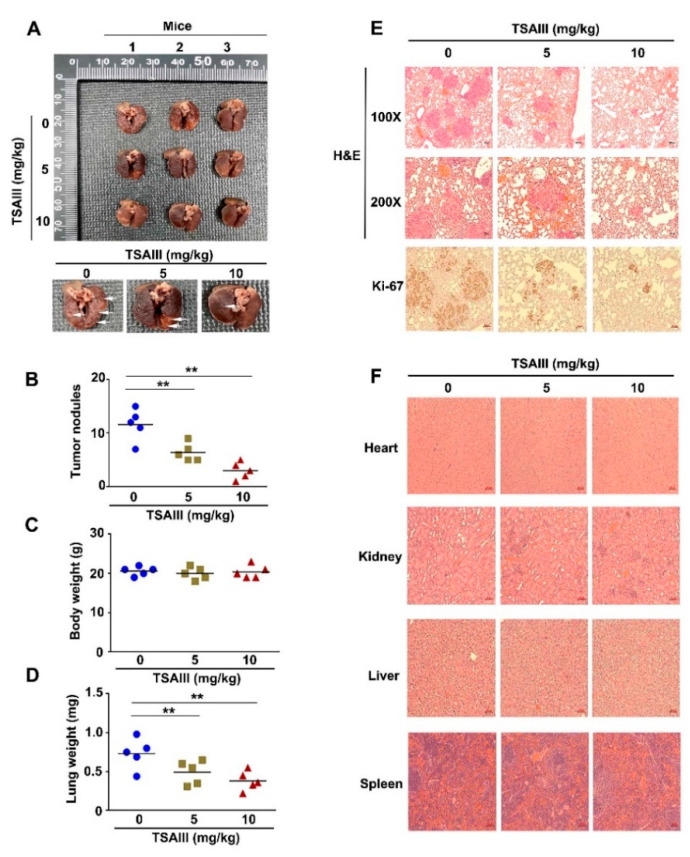
Effects of TSAIII on cervical cancer metastasis in immunodeficient mice. In animal assay of lung metastasis, human cervical cancer cells (SiHa) were harvested and injected into the tail veins of immunodeficient, female BALB/c nude mice. Mice were then administered with TSAIII (5 and 10 mg/kg) through oral gavage. After 1 month, mice were sacrificed and analyzed to observe (**A**) lung metastasis of mice. (**B**) The number of lung metastasis nodules, (**C**) body weight and (**D**) lung weight of mice were counted. (**E**) The histopathology of the lungs in metastatic tumor-bearing mice was observed. The lungs of mice were fixed in neutral-buffered formalin and stained with hematoxylin and eosin (100× and 200× magnification). (**F**) Drug toxicity of TSAIII was evaluated in mice, which showed no organ damage in heart, kidney, liver and spleen. ** *p* < 0.01 vs. control group (mean ± SE; n = 5).

The tail veins of immunodeficient, five-week-old female BALB/c nude mice were injected with human cervical cancer cells (SiHa), and subsequently, the mice were treated with TSAIII (0, 5 and 10 mg/kg). The phenomenon of lung metastasis in mice was observed (Figure 8A). The number of lung metastasis nodules (Figure 8B), the body weight (Figure 8C) and the lung weight (Figure 8D) were measured, which showed that TSAIII significantly reduced the number of lung metastasis nodules and lung weight. The histopathology of the lung tissues was carried out and revealed a notable reduction in the tumor mass in mice treated with TSAIII (5 and 10 mg/kg) as compared to the control group (Figure 8E). We also examined if TSAIII could induce drug toxicity in tissues and organs of mice. Histopathology in the heart, kidney, liver and spleen of TSAIII-treated mice was carried out and revealed no TSAIII-induced toxicity and no injuries in these tissues and organs (Figure 8F).

## 4. Discussion

Cervical cancer is still one of the most common cancers in women, resulting in annual increases in new cases and deaths worldwide. Therefore, the establishment of potential treatments for cervical cancer is urgent. Novel agents are urgently needed to improve treatment outcomes. Our findings revealed the following: (I) TSAIII suppresses cell migration, cell invasion and uPA expression without influencing cell survival, cell cycle arrest and apoptosis. (II) p38 knockdown contributing to TSAIII-inhibited uPA expression and invasion activity reveals that the p38 MAPK–uPA axis mediates the anticancer properties of TSAIII. (III) The antimetastatic properties of TSAIII were further confirmed through the pulmonary metastasis in immunodeficient mice in vivo and the anticancer stemness property of TSAIII in cervical cancer stem cells in vitro.

Previous studies revealed that inhibitory effects on cell migration and invasion activity in human renal carcinoma cells [24], osteosarcoma cells [19], NSCLC cells [15] and TSAIII inhibits HGF to induce metastatic activity in MDA-MB-231 cells [18] were not due to TSAIII cytotoxicity in the dosage range from 2 to 6 µM. Zhou et al. analyzed the antiangiogenic effect of TSAIII at a dosage from 0.5 to 2 µM in endothelial cells in vitro and in zebrafish in vivo [25]. Thus, the antiangiogenic and antimetastatic effects of TSAIII were found to contribute to its antitumor ability. Some evidence showed that a high concentration of TSAIII (IC50 > 10 μM) can induce apoptosis and has an autophagy-promoting effect in various tumor cells, such as hepatoma cells (IC50 = 15.41 µM) [26], and pancreatic cancer AsPC-1 cells (IC50 = 22.1 µM) [27]. Furthermore, the concentrations of TSAIII used in the antitumor or antimetastatic studies depend on the different tumor cell types and experimental conditions. In our results, we considered that a high concentration of TSAIII > 10 μM should inhibit cell viability and induce cell apoptosis in human cervical cancer cells. Meanwhile, a high concentration significantly induces the cell cytotoxicity of normal human CerEpi cells (Figure 1B). Therefore, our results used a low concentration of TSAIII (<6 μM) due to its strong antimetastatic effect and nontoxic effect in human cervical cancer cells, and future studies will continue to explore the antimetastatic effect and molecular mechanism of TSAIII against cervical cancer cells.

p38 MAPK is a member of the mitogen-activated protein kinases (MAPKs). Activation of the p38 MAPK pathway has been demonstrated to act as a critical factor in regulating cervical cancer through the promotion of cell proliferation and cell detachment processes [28,29]. Blockage of the p38 MAPK pathway is considered to be a potential therapeutic approach in human cancer cells [30]. A large amount of evidence emphasizes that the suppression of tumor cell migration and invasion via uPA inhibition is mediated by decreasing the phosphorylation of the p38 MAPK-dependent pathway in HCC cells [31], cervical cancer cells [32] and osteosarcoma U-2OS cells [33]. In our study, we found that TSAIII inhibited uPA expression and inactivated p38 MAPK, which is involved in the reduction of cervical cancer cell migration and invasion. In particular, we found that the cotreatment of TSAIII and p38 siRNA significantly inhibits uPA expression and the capacity for migration and invasion (Figure 6). This means that the suppression of uPA by TSAIII may be involved in the inactivation of p38 MAPK. To our knowledge, uPA is reported to be useful for predicting the metastatic potential of human cervical cancer tissues [34], and thus, targeting uPA expression might be a therapeutic strategy for human cervical cancers. As shown in Figure 4, TSAIII inhibits the protein and mRNA expression of uPA in both cervical cancer cells, implying that TSAIII-induced uPA downregulation might be caused by transcription and translation mechanism regulation. It is well known that some transcription factors are responsible for the transcription activity of uPA in various tumor cells, such as Sp1, AP1 and HOXA5 [11,35,36]. Additional studies and more experiments are needed to clarify whether some crucial transcription factors regulate the uPA promoter activity in cervical cancer cells and promote tumor metastasis.

Cervical cancer stem cells (CCSCs) are a small subpopulation of tumor cells, have a high self-renewal ability and can differentiate into heterogeneous types of tumor cells. They are important in the prognosis of patients with cervical cancer [37]. Some evidence suggests that there are high levels of Oct3/4, CD49f, Nanog and Sox2 in sphere-forming CSC-like SiHa and HeLa cells, which show higher radioresistance and tumorigenicity [38,39]. Similarly, our results indicated that TSAIII inhibits the self-renewal ability, 3D sphere formation, and CSC marker expression of the CSC-like SiHa cells in vitro (Figure 7). Therefore, using TSAIII to target CSCs may be an effective anticancer strategy against human cervical cancer. However, no studies have yet reported on the use of TSAIII in combination with chemotherapy/target therapy to treat CCSCs. In the future, more studies are needed on TSAIII-combined therapies in vitro and in vivo, specifically in CCSCs. Based on all the above-mentioned findings, these studies are expected to reveal the inhibitory effect of TSAIII on cell migration and invasion in human cervical cancer cells and provide the molecular mechanism of the TSAIII inhibition of p38 MAPK-mediated uPA expression. Therefore, TSAIII could be considered a candidate chemopreventive agent and effective therapeutic approach for the treatment of highly metastatic cervical cancer.

## 5. Conclusions

In this study, we observed that TSAIII shows antimetastatic effects in both human cervical cancer cells and CCSCs through the downregulation of p38 MAPK–uPA axis activation in vitro and in vivo. Our results suggest that TSAIII possesses a specific anticancer ability and potentially acts as a potent auxiliary antimetastatic agent against cervical cancer.

## Data Availability

Not applicable.

## Supplementary Materials

The following are available online at https://www.mdpi.com/article/10.3390/cancers15010037/s1, Figure S1: Full pictures of the Original Western blots and the densitometry scans.
