# Supplementary figures and images for: Timosaponin AIII Inhibits Migration and Invasion Abilities in Human Cervical Cancer Cells through Inactivation of p38 MAPK-Mediated uPA Expression In Vitro and In Vivo
Source: Cancers (Basel). 2022 Dec 21;15(1):37. doi: 10.3390/cancers15010037 (PMC9817900; doi:10.3390/cancers15010037)

Fig-4

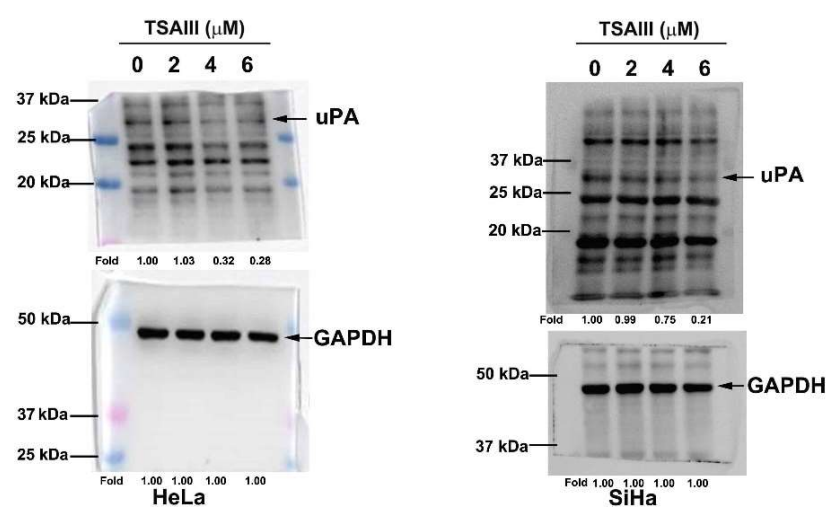

Fig-5

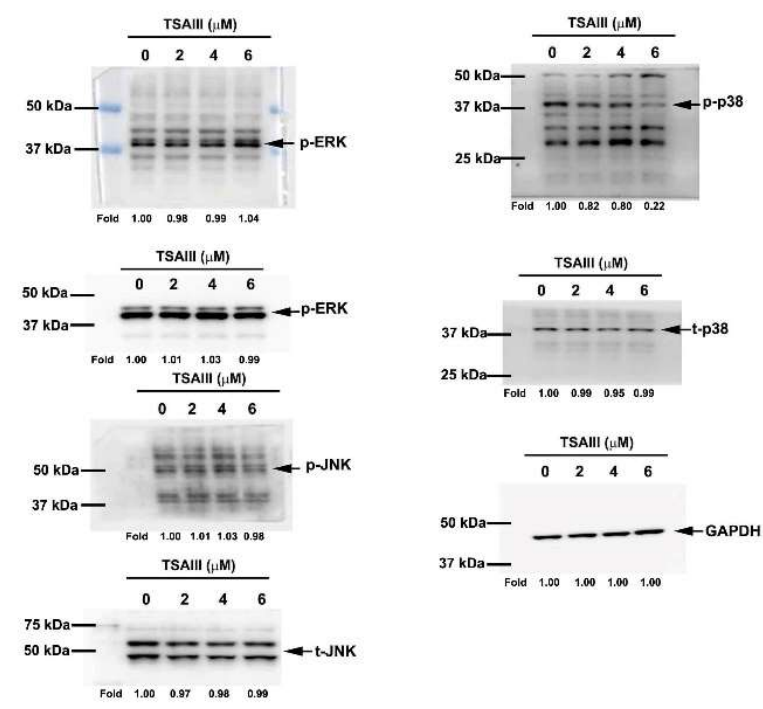

Fig-6

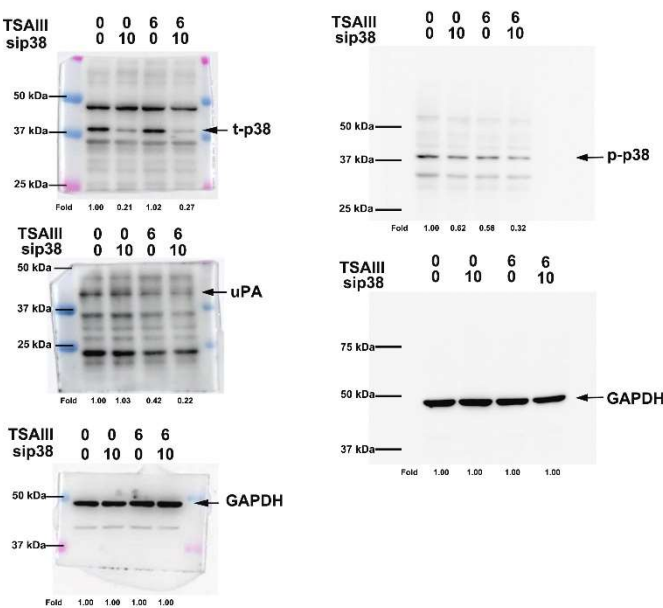

Fig-7B

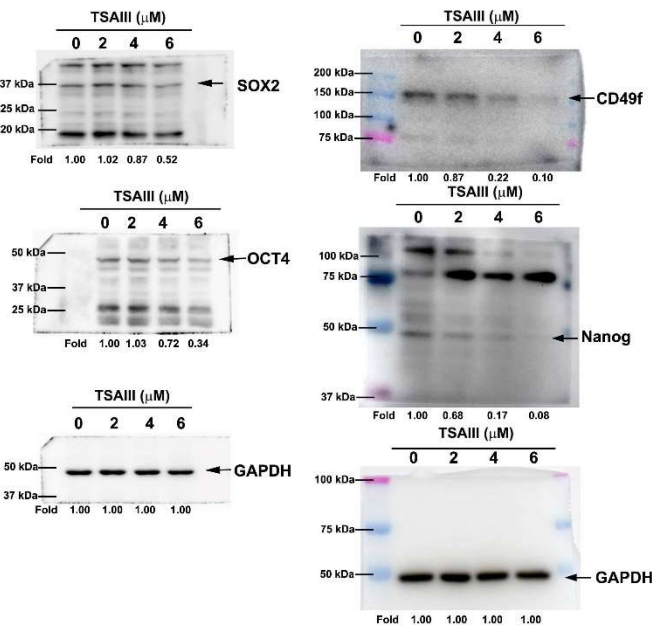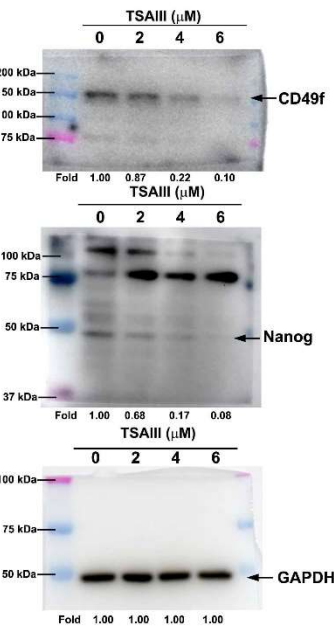

Fig-7E

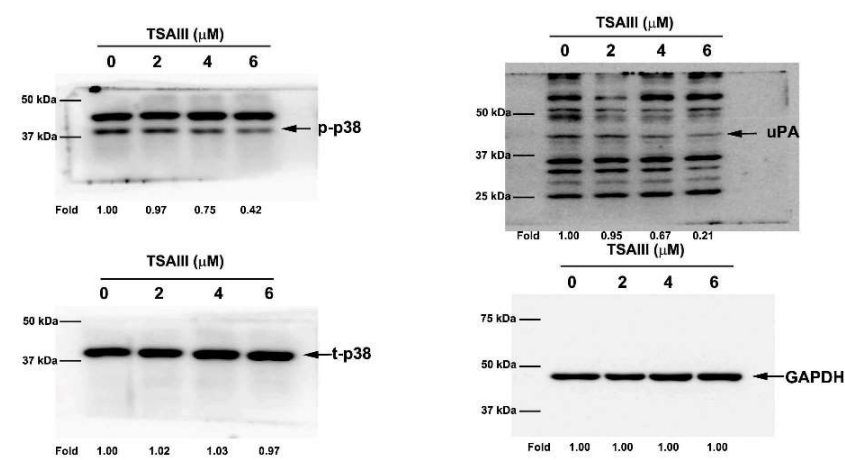

Supplement: Supplementary file 1 [file cancers-15-00037-s001.zip › Figure S1.pdf]
